# Surgical treatment of heart failure: a hot topic

**DOI:** 10.1590/S1516-31802011000300001

**Published:** 2011-05-05

**Affiliations:** I MD, PhD. Associate Professor, Discipline of Thoracic Surgery, Instituto do Coração (InCor), Hospital das Clínicas, Faculdade de Medicina, Universidade de São Paulo (HCFMUSP), São Paulo, Brazil.; II Chief Biologist, Discipline of Thoracic Surgery, Instituto do Coração (InCor), Hospital das Clínicas, Faculdade de Medicina, Universidade de São Paulo (HCFMUSP), São Paulo, Brazil.

Over recent decades, the alarming increase in the incidence of heart failure has led to recognition that there is a veritable epidemic of this disease in the Western world.^1,2^ The incidence of heart failure has been estimated to be 2% in the United States and Europe, and it may reach 13% among the elderly population.^3^

Around 10% of the patients with heart failure progress to more severe forms of the disease. These are patients who, despite optimized clinical treatment, continue to present symptoms. With disease progression, their quality of life deteriorates and their mortality rate is high.^4-6^

In Brazil, the main etiology for heart failure is chronic ischemic heart disease in association with high blood pressure. However, many diseases may lead to the final stage of heart failure, such as idiopathic dilated myocardiopathy, valve diseases, metabolic and inflammatory disorders.^7^ The final outcome for most of these diseases is heart remodeling, characterized by myocyte hypertrophy and dilatation of the cavities, thus leading to a more spherical shape for the left ventricle and reduction of the ejection fraction.^7^

Thus, proposals for new therapies and improvements in the existing therapies have been seen, resulting in significantly better treatment and management for heart failure patients. Among these techniques, the development of new medications and pharmacological regimens,^8,9^ cardiac resynchronization therapy,^10^ mechanical devices to assist the circulation, cell therapy,^11^ cardiovascular rehabilitation programs with exercises^4^ and thoracic sympathectomy,^12^ among others, can be highlighted. Within this context, heart surgery has an important role.^13^

Heart transplantation is still the best option, through providing increased quality of life and longer survival.^14,15^ This procedure has been presenting results of greater consistency, thanks to improvements and adaptations in recipient selection, donor maintenance, new immunosuppressant drugs, advances in diagnosing rejection and experience of post-transplant management.^9,16^ Nonetheless, transplantation presents many limitations, including the need for immunosuppressive therapy, high costs, donor shortage, high mortality in the waiting lists and very rigid indication criteria.

In parallel, devices for mechanically assisted circulation have been gaining space over recent years. These devices may be indicated with the aim of helping a debilitated heart to pump blood and can be used as bridging prior to transplantation, which gives such patients the chance to safely wait for a compatible donor. They can be used for recuperation therapy, in which the device not only maintains the patient's life in critical situations but also allows recovery of cardiac function. In these cases, the device can subsequently be removed. Lastly, these devices can be used as destination therapy, in which they are applied in a permanent manner when the patient presents contraindications against heart transplantation.^17-19^

The use of assisted circulation devices has become well established internationally. However, in Brazil, only limited experience with these devices has been acquired. There have been few clinical cases or experimental studies, despite the existence of large numbers of patients who would benefit from such therapy.^18-24^ The main barriers against wider use of these devices are their high cost and the almost absolute dependence on products on the international market.^19^

One of the principal characteristics of chronic heart failure is dilatation of the left ventricle, with progressive dilatation of the mitral ring and displacement of the papillary muscles. The presence of mitral failure in heart failure cases is a predictive factor for worsened quality of life and mortality.^25,26^ Several studies have demonstrated that correcting the mitral failure in patients with severe left ventricular dysfunction, either through valvuloplasty or valve replacement with preservation of the subvalvular apparatus, is associated with low mortality due to the operation, symptom relief and improved short and medium-term survival.

Disorders of intraventricular conduction are present in around 25-50% of individuals with heart failure, and left-branch block is the most frequent of these.^27^ Within such scenarios, cardiac resynchronization therapy has been used as adjuvant treatment in patients with heart failure who are refractory to optimized drug therapy.^27,28^ Diminished mortality, improved symptoms and improved quality of life, along with cardiac remodeling, have been reported by several authors, resulting from using cardiac resynchronization therapy for individuals with health failure.^10,27^ However, because of the high cost and the fact that therapeutic failure occurs in around one third of the patients undergoing this intervention, several investigations have been conducted with the aim of identifying patients who might have greater benefit from this procedure.^10^

The purpose of reverse remodeling surgery on the left ventricle is to reduce the volume of the left ventricular cavity and reestablish its shape prior to remodeling, together with complete revascularization.^29^

More recently, within our setting, left sympathetic therapeutic block by means of thoracoscopy in patients with heart failure has shown promising results.^12^ The clinical improvement and the better left ventricular function observed in patients undergoing this procedure over the first six months of follow-up have been similar to those documented from using other well established procedures for treating chronic heart failure. Findings from imaging examinations using 123I-metaiodobenzylguanidine have indicated that bilateral upper thoracic sympathectomy mildly suppresses the activation of the sympathetic nerve system, like in beta-blocker therapy.

Other procedures such as partial left ventriculectomy and dynamic cardiomyoplasty have shown controversial results and have not become established as widely used or easily accepted procedures for treating heart failure.^9^

Within this context, the natural history of heart failure has given rise to highly unfavorable prognoses and low quality of life among such patients. The search for methods that provide alternatives or are complementary to drug treatment, and which might modify the course of heart failure, is one of today's major challenges within cardiology, if not the greatest challenge, and is a hot topic within the field of cardiovascular surgery.^9,13^
